# Evolution of *HLA-B* Pharmacogenomics and the Importance of PGx Data Integration in Health Care System: A 10 Years Retrospective Study in Thailand

**DOI:** 10.3389/fphar.2022.866903

**Published:** 2022-04-05

**Authors:** Napatrupron Koomdee, Chiraphat Kloypan, Pimonpan Jinda, Jiratha Rachanakul, Thawinee Jantararoungtong, Rattanaporn Sukprasong, Santirhat Prommas, Nutthan Nuntharadthanaphong, Apichaya Puangpetch, Maliheh Ershadian, Shobana John, Mohitosh Biswas, Chonlaphat Sukasem

**Affiliations:** ^1^ Division of Pharmacogenomics and Personalized Medicine, Department of Pathology, Faculty of Medicine Ramathibodi Hospital, Mahidol University, Bangkok, Thailand; ^2^ Laboratory for Pharmacogenomics, Somdech Phra Debaratana Medical Center (SDMC), Ramathibodi Hospital, Bangkok, Thailand; ^3^ Unit of Excellence in Integrative Molecular Biomedicine, School of Allied Health Sciences, University of Phayao, Phayao, Thailand; ^4^ Division of Clinical Immunology and Transfusion Science, Department of Medical Technology, School of Allied Health Sciences, University of Phayao, Phayao, Thailand; ^5^ Department of Pharmacy, University of Rajshahi, Rajshahi, Bangladesh; ^6^ Pharmacogenomics and Precision Medicine, The Preventive Genomics and Family Check-up Services Center, Bumrungrad International Hospital, Bangkok, Thailand; ^7^ MRC Centre for Drug Safety Science, Department of Pharmacology and Therapeutics, Molecular and Integrative Biology, Institute of Systems, University of Liverpool, Liverpool, United Kingdom

**Keywords:** HLA-B, PGx, pharmacogenetics, thailand, adverse drug reactions, incorporating PGx data

## Abstract

**Background:** The *HLA-B* is the most polymorphic gene, play a crucial role in drug-induced hypersensitivity reactions. There is a lot of evidence associating several risk alleles to life-threatening adverse drug reactions, and a few of them have been approved as valid biomarkers for predicting life-threatening hypersensitivity reactions.

**Objectives:** The objective of this present study is to present the progression of *HLA-B* pharmacogenomics (PGx) testing in the Thai population during a 10‐year period, from 2011 to 2020.

**Methods:** This was a retrospective observational cohort study conducted at the Faculty of Medicine Ramathibodi Hospital. Overall, 13,985 eligible patients who were tested for *HLA-B* risk alleles between periods of 2011–2020 at the study site were included in this study.

**Results:** The *HLA* PGx testing has been increasing year by year tremendously, 94 *HLA-B* testing was done in 2011; this has been raised to 2,880 in 2020. Carbamazepine (*n* = 4,069, 33%), allopurinol (*n* = 4,675, 38%), and abacavir (*n* = 3,246, 26%) were the most common drugs for which the *HLA-B* genotyping was performed. HLA-B*13:01, HLA-B*15:02 and HLA-B*58:01 are highly frequent, HLA-B*51:01 and HLA-B*57:01 are moderately frequent alleles that are being associated with drug induced hypersensitivity. HLA-B*59:01 and HLA-B*38:01 theses alleles are rare but has been reported with drug induced toxicity. Most of the samples were from state hospital (50%), 36% from private clinical laboratories and 14% from private hospitals.

**Conclusion:** According to this study, *HLA-B* PGx testing is increasing substantially in Thailand year after year. The advancement of research in this field, increased physician awareness of PGx, and government and insurance scheme reimbursement assistance could all be factors. Incorporating PGx data, along with other clinical and non-clinical data, into clinical decision support systems (CDS) and national formularies, on the other hand, would assist prescribers in prioritizing therapy for their patients. This will also aid in the prediction and prevention of serious adverse drug reactions.

## Introduction

ADRs are any unrelated or unexpected reactions to drugs that have been approved for normal use in normal dosage (Cameron and Ramsay, 1984). When it becomes serious, unpredictable, and life threatening, it becomes a public concern. Another significant problem with ADRs are the financial burden and longer hospital stays. According to data from the United States (1966–1996), 6.7 people out of every 100 patients have severe ADRs (Lazarou et al., 1998; Chyka, 2000), whereas in China, severe ADRs account for 10% of the 1.676 million ADRs complaints (CDR-ADR, 2021). In the case of Thailand, ThaiVigibase received around 600,000 reports between 1984 and 2014, with over half of them reported in the last 6 years and significant ADRs accounting for 20% of all reports (Genome, 2012).

Pharmacovigilance is the science of detecting, assessing, and comprehending ADRs signals in order to prevent ADRs or other drug-related problems in the future (DRPs) (Edwards and Aronson, 2000). Throughout the drug development process, a variety of approaches are used to predict and assess ADRs. Safety pharmacology profiling (*in vitro* biochemical and cellular assays) and computational methods, such as protein target-based and chemical structure-based approaches, were used in the preclinical stage (Whitebread et al., 2005). In different stages of clinical trial, the safety is monitored only in few people. Only during post-marketing surveillance, through spontaneous ADRs reporting, observational case-control, and cohort studies, can meaningful ADRs data be collected.

PGx is an important data source for customizing treatment and predicting and preventing ADRs. In the last 10 years, ADRs-pharmacogenetics has moved beyond just identifying risk alleles to developing different guidelines in different nations based on the clinical relevance of those risk genes. The comprehensive integration of this genetic data into clinical practice, however, has remained a considerable challenge. The reason could be a lacuna in the integration of various biological data with clinical efficacy and ADRs. However, integrative approaches to ADRs prediction have recently gained popularity. A new computational framework was developed to predict ADRs by Huang et al., in which the biological data such as protein-target interaction, protein-protein interaction, gene ontology, annotation, and reported ADRs were integrated (Huang et al., 2011). Recently, studies have shown the integration of phenotypic information with chemical and biological properties of drugs to predict ADRs (Liu et al., 2012a).

However, integration of preclinical study data with ADRs may not be that effective because it is well known that the pharmacodynamics of a drug is a complex phenomenon, especially with regard to its on-target and off-target interactions. Hence, it is important to develop one framework to understand the ADRs by integrating various types of data into it (Liu et al., 2012b). The various types of data include biological, genetic, pharmacokinetic, and other clinical and non-clinical data.

Human leukocyte antigen (*HLA*) variations and their relationship to drug-induced severe hypersentivity reactions are intensively investigated in ADRspharmacogenetics in various countries, especially in Thailand. Carbamazepine (Phillips et al., 2018), oxcarbazepine (Leckband et al., 2013), phenytoin (Karnes et al., 2020), allopurinol (Hershfield et al., 2013), and abacavir (Martin et al., 2012a) are the five medications for which *Clinical Pharmacogenetics Implementation Consortium* (CPIC) PGx implementation recommendations are now available. Despite the fact that Thailand is the first country in East Asia and the fourth in Asia to publish a large number of PGx research papers (Ang et al., 2017), this is the first cohort study in Thailand to use more than 10,000 data to report the evolution of *HLA-B* PGx testing for various drugs. The goal of this study is to present the 10 years *HLA-B* data and to recommend the importance of incorporating PGx data into clinical decision support systems (CDS), particularly in the prevention of severe cutaneous adverse drug reactions (SCARs).

## Materials and Methods

### Study Design/Setting

**FIGURE 1:**
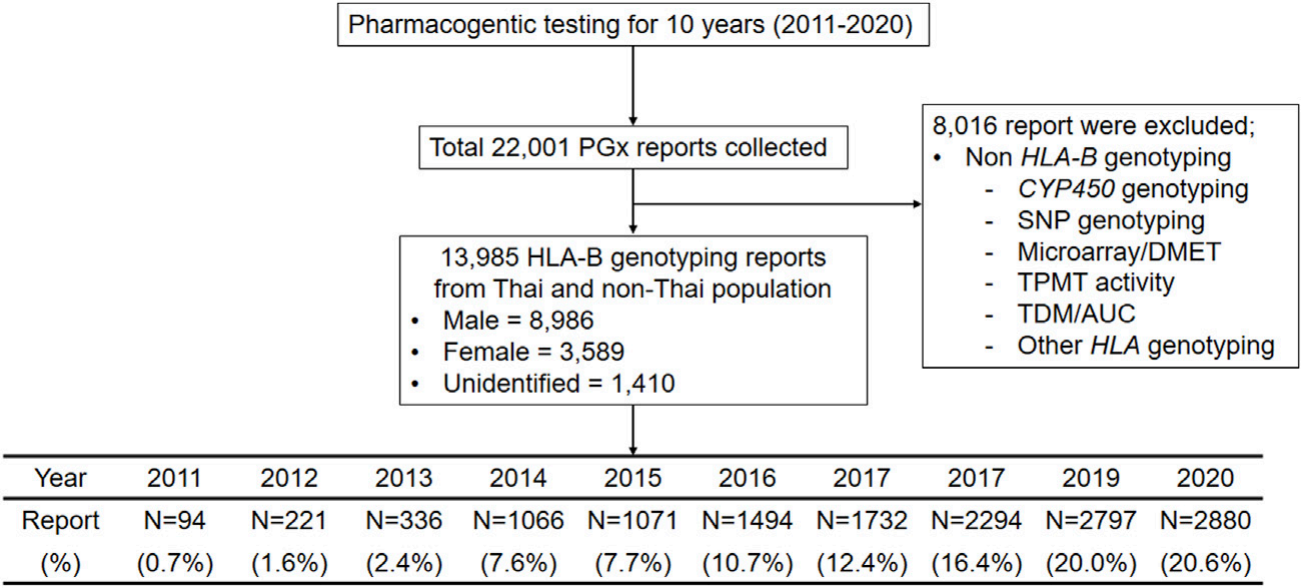
Flow chart of Clinical pharmacogenomics study designed, inclusion and exclusion criteria for 10 years during 2011–2020. This flow diagram illustrates the pharmacogenetic reports collected in this study, the number of reports (N = 22,001) and inclusion and exclusion criteria.

This was a cohort study that was conducted retrospectively. All PGx data was obtained from the Pharmacogenomics and Personalized Medicine laboratory (PPM) at the Somdech Phra Debaratana Medical Center (SDMC), Faculty of Medicine Ramathibodi Hospital, Bangkok Thailand (N = 22,001). A descriptive, observational, cross-sectional, retrospective study was conducted with 13, 985 *HLA-B* genotyping reports during 2011–2020 (Figure 1). This study was approved by the Ethical Review Committee on Research Involving Human Subjects, Faculty of Medicine, Ramathibodi Hospital, Mahidol University (ID 04-56-42). The Strengthening the Reporting of Observational Studies in Epidemiology (STROBE) reporting guideline was followed for cohort studies in this study (Von Elm et al., 2008).

### Data Selection

The *HLA-B* PGx testing approach to prescribing medications was represented by this cohort. The PPM provided a total of 22,001 PGx testing results. This study includes 13,985 eligible patients who were treated with drugs such as allopurinol, abacavir and carbamazepine etcfrom all Out-Patient Department (OPD)/In-Patient Department (IPD) and screened for *HLA-B* gene or risk alleles. Patients or samples who had their *HLA-B* gene or alleles tested only at our lab were included, but no restriction was kept from including the samples from different sources in Thailand. For example, the samples came from throughout the country, from private and public hospitals. The samples received from lab centers that serve many hospitals and clinical sectors in Thailand were also included. We have included all the *HLA-B* genetic biomarkers regardless of the availability of the CPIC recommendations. Patients tested for genetic variants of *CYP450,* other genes, and therapeutics drug monitoring (TDM), on the other hand, were not included in the study (N = 8,016).

### *HLA-B* Genotyping

The polymerase chain reaction-sequence-specific Oligonucleotide probe (PCR-SSOP) test and LuminexTM Multiplex Technology were used to analyze *HLA-B* alleles according to well-established protocols. Briefly, the PCR products were hybridized against a panel of oligonucleotide probes coated on polystyrene microspheres. The probe sequences were complementary to polymorphic sequence stretches within the target *HLA-B* alleles. A colorimetric reaction and fluorescence detection technology were used to visualize the amplicon-probe complex. *HLA* fusion™2.0 software was used to analyze data from the *HLA-B* assays. The discovered alleles can be accurately reported at the 2-field or 4-digit level using the common intermediate well document (CIWD; version 3.0.0: common, intermediate, and well-documented *HLA* alleles in world populations) 2020. A recommended CIWD list is made up of the most prevalent category in the total or any of the seven geographic/ancestral/ethnic groups.

### Variables and Data Analysis

This study examines the progress of *HLA-B* PGx testing in the Thai population during a 10 years period, from 2011 to 2020. Based on this 10 years data, the number of *HLA-B* tests performed each year, the most prevalent drugs for which *HLA-B* genotyping was requested, the *HLA-B* allele and carrier frequency were reported. In addition, the paper analyzed the sources of samples and the frequency of common PGx biomarkers that have a strong correlation with various ADRs.

Descriptive statistics were employed to summarize the collected data and applied to analyze the genotyping data. The *HLA-B* allele frequencies of the samples were assayed by direct counting and, subsequently, by dividing the total number of occurrences of that allele by the total number of alleles at that locus in the population.

## Results

### Pharmacogenomics(PGx) Testing in Thailand

A total of 13,985 patients were found to be eligible from the 22,001 data reviewed, including 8,986 males and 3,589 females (Figure 1). The reports were excluded from this analysis if there were non-*HLA-B* genotyping such as *CYP450* genotyping, microarray for drug metabolism enzyme and transpoter genes (DMET), Thiopurine S-methyltransferase (TPMT) activity and therapeutic drug monitoring/area under the curve (TDM/AUC).

**FIGURE 2:**
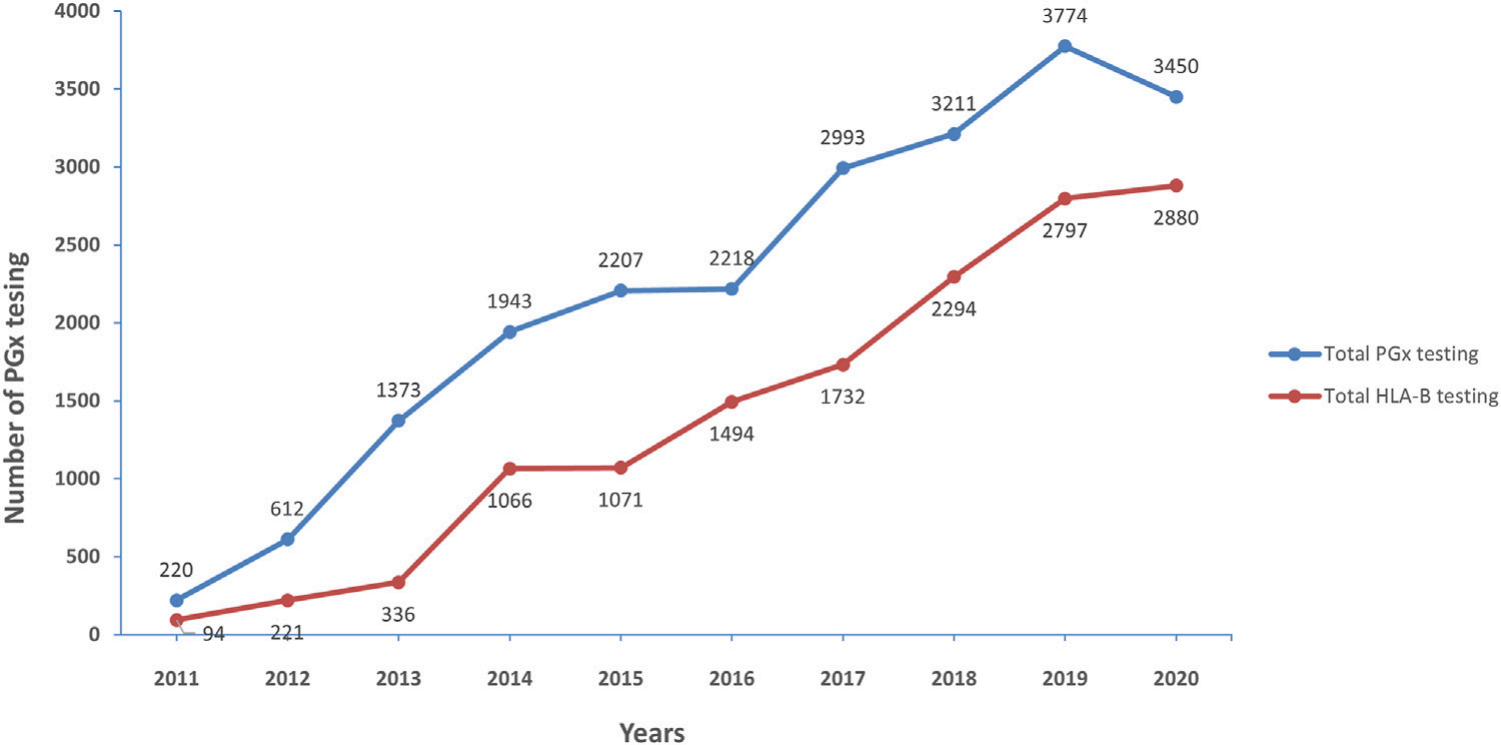
Number of pharmacogenetic testing for 10 years (2011–2020), the increase of pharmacogenetic testing (blue line) and *HLA-B*-pharmacogenetic testing (red line) over 10 years during 2011–2020.

Figure 2 illustrates the trend of PGx testing from 2011 to 2020 in Thailand. Totally, 22,001 PGx testing were performed in the last 10 years. Interestingly, only 220 samples were requested by clinicain, but the number of PGx testing was escalated to 3,450 in the 2020. This demonstrates that PGx testing has been utilized in clinical practice of Thailand routinely.

The similar trend was found in *HLA-B* PGx testing; it started with 94 tests in 2011 and has gradually risen every year, with the greatest number of tests reported in 2020 with 2,880 tests. The most common *HLA-B* genotyping was requested before prescription of allopurinol, abacavir, and carbamazepine.

### *HLA-B* Allele and Carrier Frequency in Thai Population

Among the total of 13,985 *HLA-B* genotyped data, 1,560 report were identified as non-Thai. Therefore, the allele and carrier frequencies of *HLA-B* alleles in the 12,425 Thai population are presented in supplement table 1S.This information is based on 10 years of PGx testing at the PPM laboratory. Among all the listed alleles, five *HLA-B* alleles are commonly distributed among Thai population and those are HLA-B*46:01 (24.56%), HLA-B*40:01 (17.87%), HLA-B*58:01 (16.03%), HLA-B*15:02 (14.96%), HLA-B*13:01 (12.91%). Among these common alleles, HLA-B*13:01 (dapsone and co-trimoxazole), HLA-B*15:02 (carbamazepine and oxcarbazapine)*,* and HLA-B*58:01 (allopurinol) have been found to be substantially related with drug-induced toxicity in the Thai population.

The frequency of following alleles HLA-B*51:01, HLA-B*52:01, HLA-B*55:02, HLA-B*57:01, HLA-B*07:05, HLA-B*15:01 and HLA-B*44:03 was found to be in the range of 3.00-9.00%. Out of those, HLA-B*57:01 were found to be substantially linked to abacavir-induced hypersensitivity reactions. Some alleles are not presented in higher frequencies among Thai people, but they have been associated to a number of significant drug-related disorders such as HLA-B*15:08, HLA-B*38:01, and HLA-B*59:01.

The distribution of PGx markers associated with various drug induced cutaneous reactions among Thai patients is shown in Table 1. Another high-frequency allele revealed in this study was HLA-B*58:01, this frequency rate in our study is greater rate than other Thai studies. HLA-B*13:01 has been associated with SCARs induced by phenytoin, phenobarbital, dapsone, cotrimoxazole, and salazosulfa-pyridine. Carbamazepine, oxcarbazepine, and cotrimoxazole-induced Stevens-Johnson syndrome (SJS)/Toxic epidermal necrolysis (TEN) were found to be associated with HLA-B*15:02. Other *HLA-B* alleles in serotype 75, including HLA-B*15:08, HLA-B*15:11, and HLA-B*15:21, have been associated with SJS/TEN caused by carbamazepine. Some genotypes have a lesser incidence, yet they have a strong link to medication toxicity. HLA-B*59:01 and HLA-B*38:01, for example, have been associated with SJS/TEN caused by methazolamide and co-trimoxazole respectively. Since 2011, the *HLA-B* gene has been the most frequently genotyped gene, with a particularly notable increase after 2015. This constitutes 63% of the total PGx testing in our lab.

**TABLE 1:** The *HLA-B* alleles associated with cutaneous adverse drug reactions (CADRs) and carrier frequency of Thai population (N = 12,425).

*HLA*pharmacogenetics Marker	Drug	ADR Type	Carrier Frequencies (%)
HLA-B*13:01	Phenytoin	SCARs	12.91
	Phenobarbital	DRESS	
	Dapsone	SCARs	
	Co-trimoxazole	DRESS	
	Salazosulfa-pyridine	DRESS	
HLA-B*15:02	Carbamazepine	SJS/TEN	14.96
	Oxcarbazepine	SJS/TEN	
	Co-trimoxazole	SJS/TEN	
HLA-B*15:08	Carbamazepine	SJS/TEN	0.13
	Phenytoin	SJS/TEN	
	Co-trimoxazole	SJS/TEN	
HLA-B*15:11	Carbamazepine	SJS/TEN	0.60
HLA-B*15:13	Phenytoin	SJS/TEN, DRESS	1.66
HLA-B*15:21	Carbamazepine	SJS/TEN	1.09
HLA-B Serotype 75 (HLA-B*15:02, HLA-B*15:08,HLA-B*15:11, HLA-B*15:21)	Carbamazepine	SJS/TEN	16.78
HLA-B*35:05	Nevirapine	SJS/TEN, DRESS	3.72
HLA-B*38:01	Co-trimoxazole	SJS/TEN	0.22
HLA-B*38:02	Oxcarbazepine	MPE	6.94
	Co-trimoxazole	SJS/TEN	
HLA-B*51:01	Phenobarbital	SJS/TEN	7.18
HLA-B*56:02	Phenytoin	DRESS	1.01
HLA-B*57:01	Abacavir	AHS	3.33
	Flucloxacillin	DILI	
HLA-B*58:01	Allopurinol	CADRs, SCARs, MPE	16.03
HLA-B*59:01	Methazolamide	SJS/TEN	0.00

MPE, maculopapular exanthema; SCARs, severe cutaneous adverse reactions; SJS, Stevens-Johnson syndrome; TEN, toxic epidermal necrolysis; DRESS, drug reaction with eosinophilia and systemic symptoms; AGEP, Acute Generalized ExanthematousPustulosis; CF, carrier frequency; *HLA*, *human leukocyte antigen*.

### *HLA-B* Genotyping

**FIGURE 3:**
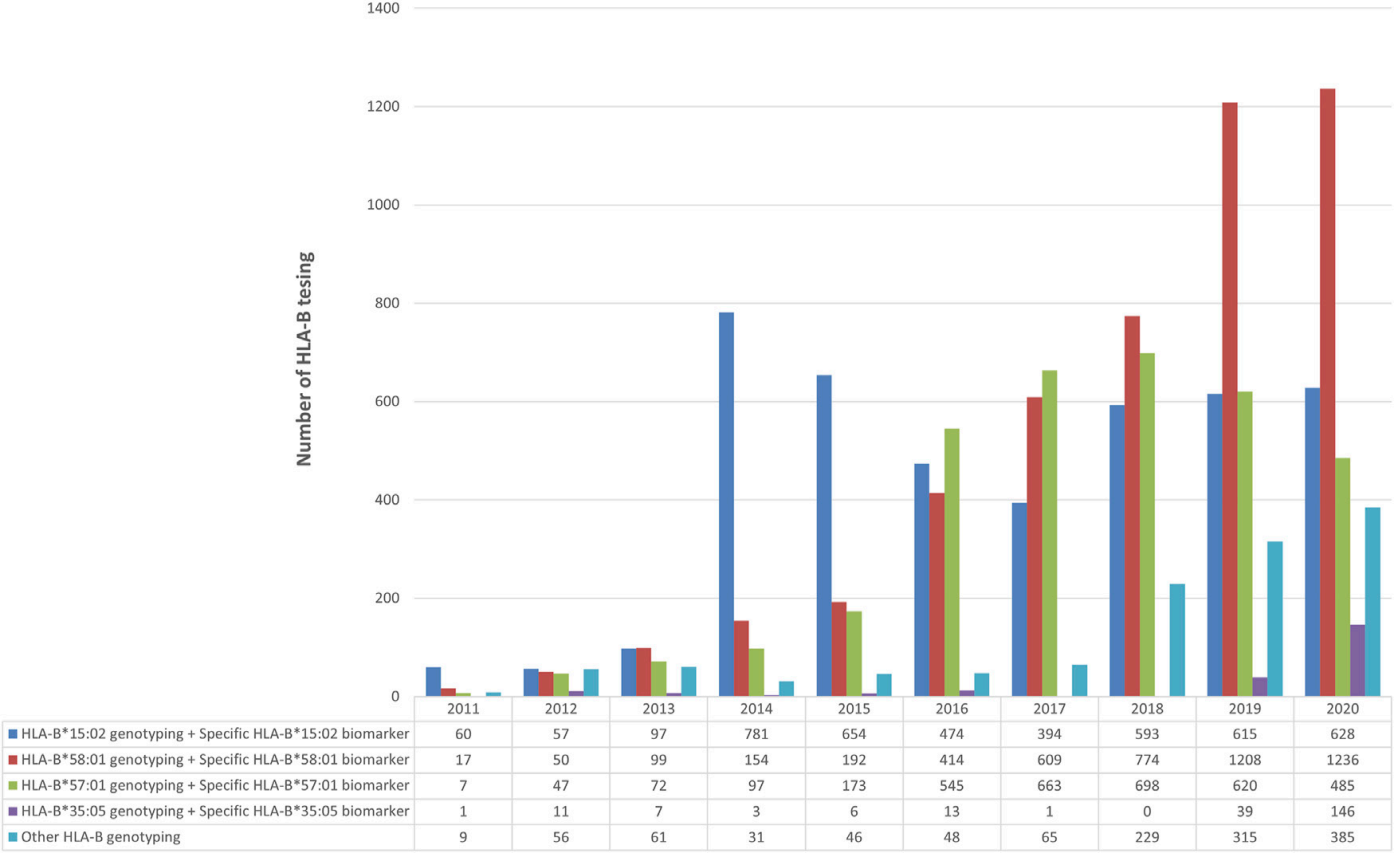
Number of *HLA-B*-pharmacogenetic testing for (1) carbamazepine (2) allopurinol (3) abacavir (4) Nevirapine and (5) other drugs over 10 years during 2011–2020.

*HLA-B* genotyping was done either a specific allele or the entire gene for drugs to determine their association with toxicity. Among all the *HLA-B* genotyping, the number of specific HLA-B*15:02 alleles or the whole *HLA-B* genetic testing for carbamazepine was higher since the beginning. However, the maximum number of tests was done in 2014; later the rate was slightly reduced but still held the second place in *HLA-B* PGx testing. The allopurinol-induced SCARs has been linked to HLA-B*58:01. The number of tests for the past 10 years has been increasing upward. Since 2017, the rate has surpassed the HLA-B*15:02 testing. In 2020, this was the most tested *HLA-B* allele in our lab (*n* = 1,236). *HLA-B* and specific HLA-B*57:01 were tested for abacavir-induced hypersensitivity reactions. In 2017 and 2018, its testing rate was higher than HLA-B*15:02. Other than these alleles, recently the number of nevirapine and HLA-B*35:05 association tests was higher than in any other year (Figure 3).

**FIGURE 4:**
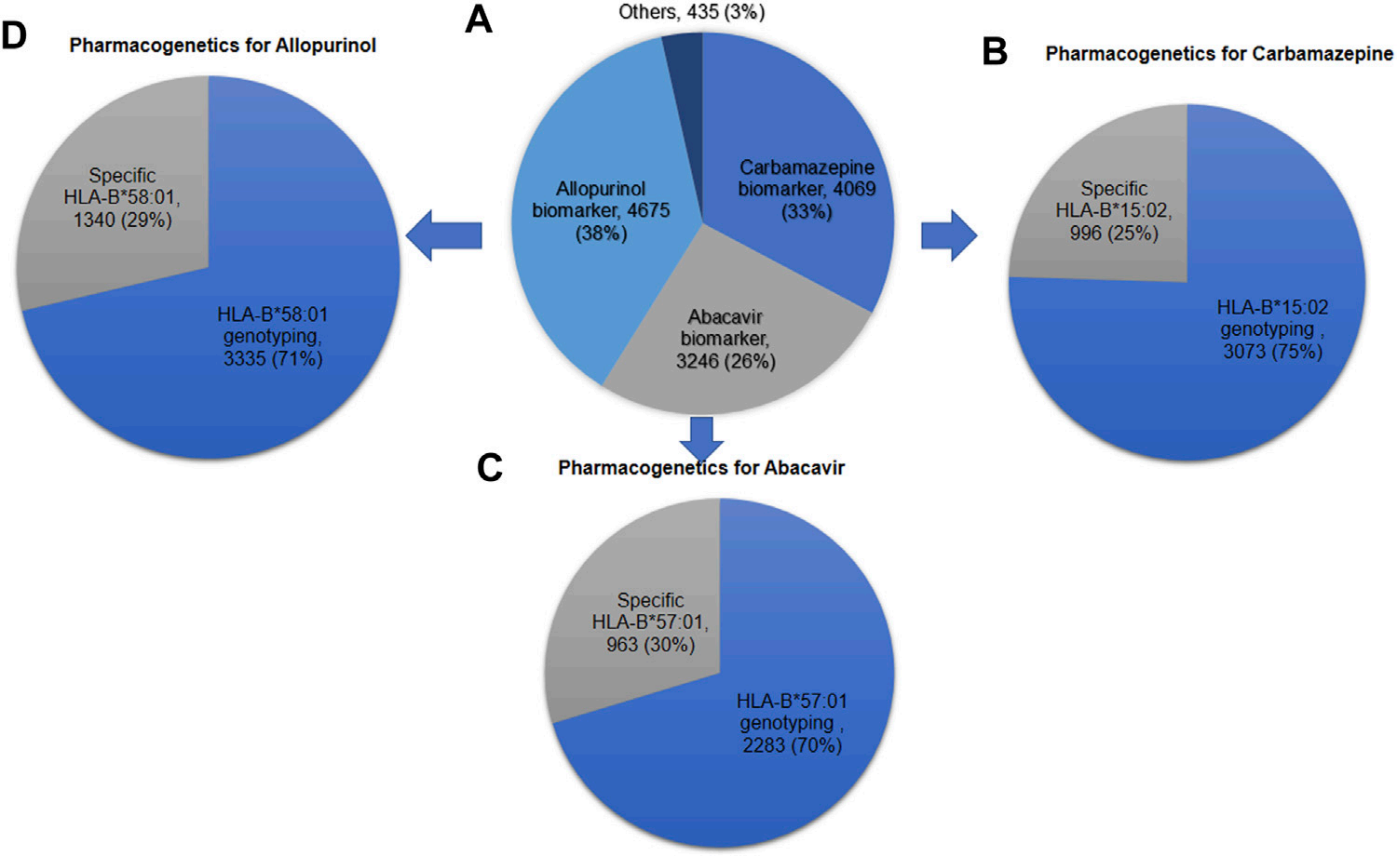
Number of *HLA-B* pharmacogenetic testing requested for Thai patients to prevent severe cutaneous adverse drug reactions over 10 years during 2011–2020 **(A)** *HLA-B* pharmacogenetic testing for all drugs (N = 12,425); **(B)** Pharmacogenetic testing for carbamazepine (N = 4,069); **(C)** Pharmacogenetic testing for abacavir (N = 3,246); **(D)** Pharmacogenetic testing for allopurinol (N = 4,675).

Figure 4 shows the number of *HLA-B* pharmacogenetic testing requested for Thai patients to prevent SCARs over 10 years during 2011–2020. Specific HLA-B*15:02 allele was genotyped less frequently than the entire *HLA-B* geneto prevent carbamazepine induced SCARs. For example, out of the 4,069 total carbamazepine samples analyzed, 75% requested the *HLA-B* gene tested, whereas the remaining 25% requested the HLA-B*15:02 variant tested alone. Similarly, 29% of the 4,675 samples asked for the HLAB*58:01 alleleto be tested. In abacavir, the particular allele, HLA-B*57:01, was tested in 30% of the samples.

## Discussion

### *HLA-B* Variants and Their Association With SCARs

This cohort study showed the PGx data of *HLA* gene and its association with various drug induced ADRs. In our investigation, the HLA-B*46:01 allele was shown to be more prevalent (24.56%) but its association with any ADRs not significant. However, this allele was identified as a risk factor for cutaneous adverse drug reactions (CADRs) in a recent study conducted among Han Chinese (Deng et al., 2017). Another high-frequent allele revealed in our study was HLA-B*58:01, which has a greater rate than (16.33%) other Thai studies (Tassaneeyakul et al., 2009). This could be because our results are based solely on PGx testing done in our facility. This allele distribution is higher among central Africans (10–15%), and it ranges from 5 to 15% in India (Hughes et al., 2004). In the present study, we found that 16.33% of Thai people carry the HLA-B*58:01 allele, which is associated with allopurinol hypersensitivity in gout treatment (Saokaew et al., 2014; Ueta et al., 2014). For example, the strength of the association was higher in the Han Chinese (100%) (Hung et al., 2005), Thai (100%) (Tassaneeyakul et al., 2009), and Korean populations (80%) (Kang et al., 2011) than in the Japanese (55%) (Kaniwa et al., 2008), and European populations (56%) (Lonjou et al., 2008).

HLA-B*15:02 was the next most prevalent allele which considered to be the known valid pharmacogenetic biomarker for carbamazepine induced SJS/TEN. Chung W H et al. was first reported its association with carbamazepine-SJS/TEN in Taiwanese Han Chinese (Chung et al., 2004). This association was later confirmed in several other populations, including Chinese (Wang et al., 2019), Thai (Tassaneeyakul et al., 2010), Malaysians (Chang et al., 2011), Indians (Mehta et al., 2009), Vietnamese (Nguyen et al., 2015), and Indonesians (Yuliwulandari et al., 2017). A recent meta-analysis study which included 11 studies of Chinese, Korean and Thai populations revealed a strong association between HLA-B*15:02 and lamotrigine induced SJS/TEN in the Chinese population (OR = 2.4) (Deng et al., 2018). The association between HLA-B*15:02 and oxcarbazepine-induced SJS/TEN has also been demonstrated by Hung S I et al., 2010. In the same serotype B 75, HLA-B*15:21 has shown positive association with carbamazepine induced SJS, this has been confirmed many other Asian population such as Thai (Jaruthamsophon et al., 2017) Indonesian (Khor et al., 2014; Yuliwulandari et al., 2017) and in Filipinos (Capule et al., 2020). Other than these alleles, in *HLA-B* 15 family, HLA-B 15:11, HLA-B*15:13, HLA-B*15:08 were found to be the risk factors for drug induced hypersensitivity reactions in various populations (Kaniwa et al., 2010; Chang et al., 2017).

Our study reveals that 13% of the population carry HLA-B*13:01. In Chinese, the frequency of HLA-B*13:01 ranges between 3 and 8% across North and South China, but the people of Papua New Guinea and Melanesians and Australian Aboriginals who are evolutionarily related to Papuans, to have the highest reported allele frequency in the world (28%) (Gonzalez-Galarza et al., 2011)**.** This allele was initially discovered as a risk factor and predictor of drug hypersensitivity syndrome (DHS) in the Chinese population (Zhang et al., 2013). HLA-B*13:01 has been shown in multiple other studies in Asia as being a strong predictor of the dapsone induced DHS (Chen et al., 2018; Tangamornsuksan and Lohitnavy, 2018). However, few studies reported its association with Aromatic antiepileptic drugs (AEDs) induced hypersensitivity reactions (Shi et al., 2011; Min et al., 2019).

Abacavir is a prodrug, and a nucleoside reverse transcriptase inhibitor (NRTI) used for the treatment of HIV. It elicits DHR in up to 8% of the population, with drug hypersensitivity reactions (DHR) attributed to the prodrug itself. Fever, malaise, nausea, vomiting, rashes are the clinical features of abacavir-induced DHR. In 2002, HLA-B*57:01 was found to be associated with abacavir induced DHR in the North American population, and later on, this association was confirmed in the Australian and United Kingdom populations (Hetherington et al., 2002; Mallal et al., 2002; Hughes et al., 2004). However, this association was not noticeable among black people. The reason could be the lesser prevalence of this allele in this population (2.5%) (Saag et al., 2008) European population (6–7%). This allele frequency among Asians is found to be around 12.6% (Martin et al., 2012b). However, in this study, the prevalence was found to be around 3.3%. The reason could be the frequency data is based the data from our lab. This association has been well validated and is a good example of a pharmacogenetic test used widely in the clinic before drug prescription, with abacavir not now being prescribed for individuals positive for HLA-B*57:01 (Pavlos et al., 2017).

The HLA-B*51:01 allele has consistently been associated with AEDs-induced CADRs in various Asian ethnicities. Its frequency in our study was found to be 7%. In a Han-Chinese population, a relationship between carbamazepine-induced Maculopapular exanthema (MPE)/Drug Reaction with Eosinophilia and Systemic Symptoms (DRESS) and HLA-B*51:01 (OR 4.56, 95 percent CI 2.0–10.5, *p* = 0.01) was discovered (Hsiao et al., 2014). This allele association with AEDs-induced CADRs has been confirmed in North and South India (Ihtisham et al., 2019; John et al., 2021), Japan (Kaniwa et al., 2013), Thailand (Tassaneeyakul et al., 2016; Manuyakorn et al., 2020), and among Koreans (Kim. et al., 2016). As a result, in some populations where HLA-B*51:01 is more common, HLA-B*51:01 could be a phenotype-specific genetic marker for carbamazepine-phenytoin/SCARs or phenytoin-DRESS.

### Recommendations for Integration of PGx in CDS and Other National Guidelines

Because the majority of pharmaceuticals are discovered in western countries (United States, Europe, Australia, New Zealand, and Canada), therapy selection is successful in only half of the world population in most cases (Howard, 2002). Only little thought has been given about how these drugs will be used throughout the world. Individualized treatment is regarded to be the greatest choice for therapy selection, and good level therapy selection is based on geographical/ethnicity/racial population. The worst method of treatment selection is based on a common population estimate. Integrating PGx data with other clinical and non-clinical data can assist clinical practitioner in selecting the best and most appropriate treatment for their patients.

To achieve a positive therapeutic outcome, rational prescribing is critical. Incorporating PGx data into drug formularies can aid clinical decision-making in public health. Amadioquine is an anti-malarial drug whose usage has been restricted due to its hepatotoxicity. However, there is a considerable demand for this drug in Africa and Southeast Asia, where malaria is still a serious problem. However, it was only after 2005 that it was shown that patients with the CYP2C8*3 allele were linked to Amadioquine-induced hepatotoxicity (Cavaco et al., 2005). Combining PGx data with national drug formularies, particularly for drugs on the essential drug list, can aid in public health decision-making (Roederer and McLeod, 2010). It has the potential to increase physician and other health-care professionals’ awareness of PGx, as well as their confidence in using it in medical practice. It can assist clinicians in prioritizing treatment for each patient based on PGx data. This may assist the researcher in obtaining funding and infrastructural support for future PGx experiments.

However, because clinical practice differs widely between nations, discrepancies in PGx implementation guidelines is another significant issue in integrating PGx data with the Essential Drug List. When the same drug has distinct genotyping and dose recommendations, for example, this disparity causes confusion among health care providers when using clinical PGx guidelines (Guo et al., 2019). Better guidelines could be developed based on ethnic and geographic preferences.

Another step in individualized treatment and preventing severe adverse drug responses is to combine PGx data in a CDS. Every year in the United States, over 10,000 people die as a result of ADRs, despite the fact that drugs are properly prescribed and provided. According to Phillips et al., 60% of the 27 ADRs involved medications had a PGx connection (Lazarou et al., 1998; Phillips et al., 2001). Hence, every ethnic/geographical community requires a PGx clinical decision support tool. A previous study revealed the establishment of a disease-drug search engine data base, which was subsequently combined with the genomic prescribing system. The study recommended PGx in CDS would make drug prioritization easier.

Our study shows that *HLA-B* PGx testing has been increasing tremendously for the past 10 years in our lab. The general reasons could be the emerging sequencing technologies which shrinking the cost of sequencing (Schwarze et al., 2018), the better understanding or awareness about PGx application among health care professional especially among physician, and availability of standard international and national guideline on the clinical utility of PGx testing (Guo et al., 2021).

The first south East Asian country which had record breaking research and publications in this field, and scientific bodies that funding many genomic research projects such as Thailand center of excellence for life sciences (TCELS), Genomic Thailand initiatives (GTI) gives the opportunity for researcher to produce clinically valid data for each PGx biomarkers in Thai population. Since 2011, the *HLA-B* has been the most frequently genotyped gene, with a particularly notable increase after 2015. This constitutes 63% of the total PGx testing in our lab. The *CYP450* gene was the next common gene for genotyping. The maximum number of *HLA-B* tests was done in 2020 (n = 2,880). The reason could be that the Thai population that comes under the Universal Coverage Scheme (UCS) has coverage for HLA-B*15:02 screening with a reimbursement of 28 dollars per person. Our study also shows that 50% of the samples were from state hospital, this could be due to establishment of a policy that require HLA-B*15:02 pharmacogenetic testing prior to the start of carbamazepine medication by the Thai Department of Medical Sciences and the National Health Security Office (NHSO) in 2013 and 2018 respectively (Kaniwa et al., 2008).

However, there is currently no PGx alert system connected with the electronic health records (EHR) in Thailand. Failure to integrate PGx alert into the EHR system could result in major clinical consequences. For example, in Thailand, an inpatient with the HLA-B*15:02 allele was initially administered phenytoin but was later switched to carbamazepine due to a lack of a PGx alert system in the EHR, and the patient died as a result of carbamazepine-induced TEN (Sukasem et al., 2021).

Lastly, other *HLA* was reported to be associated with drug-induced SCARs, including DRESS, SJS/TEN, and AGEP (Table 2). When determined the carrier frequency from the previous study (Satapornpong et al., 2020), the frequencies of these biomarkers were common in Thai population. Especially, pharmacogenetic markers associated with different ethnic groups such as HLA-A*24:02 and HLA-A*31:01 for carbamazepine, HLA-A*33:03 and HLA-C*03:02 for allopurinol etc. Further case-control study with large sample size need to be warranted to confirm these findings in Thai population.

**TABLE 2:** The HLA alleles associated with SCARs and carrier frequency of Thai population (N = 470) from published article (Satapornpong et al., 2020).

HLA Allele	Drugs	Type of SCARs	Thailand	Ethnic Group	Ref
			CF (%)		
HLA-A*01:01	Phenobarbital	SCARs	4.468	Thai	Manuyakorn et al. (2016)
HLA-A*02:06	Cold medicine	SJS,TEN	4.468	Japanese	Ueta et al. (2014)
HLA-A*24:02	Carbamazepine	SJS,TEN,MPE	20.213	Han Chinese, Korean	(Moon et al., 2015; Shi et al., 2017)
	Lamotrigine				
	phenytoin				
HLA-A*31:01	Carbamazepine	SJS/TEN, AGEP	1.489	Caucasian, Japanese, Korean, Chinese, and patients of mixed origin	Amstutz et al. (2014)
HLA-A*32:01	Vancomycin	AGEP, DRESS,SJS/TEN	0.426	Europeans	(Cristallo et al., 2011; Konvinse et al., 2019)
HLA-A*33:03	Allopurinol	SJS,TEN	21.064	Caucasian, Asian populations	(Cristallo et al., 2011; Li et al., 2017)
HLA-A*68:01	Lamotrigine	SCARs	1.915	Europeans	Kazeem et al. (2009)
HLA-C*03:02	Allopurinol	SJS,TEN	14.68	Caucasian, Asian populations	Li et al. (2017)
HLA-C*04:01	Nevirapine	SJS,TEN	9.36	Malawian	(Carr et al., 2013; Carr et al., 2017)
HLA-C*06:02	Co-trimoxazole	SJS,TEN	8.51	Thai	Kongpan et al. (2015)
HLA-C*08:01	Carbamazepine	SJS,TEN	19.15	Han Chinese	Shi et al. (2017)
	Phenytoin				Hung et al. (2010)
	Allopurinol			Caucasian	Cristallo et al. (2011)
	Co-trimoxazole			Thai	Kongpan et al. (2015)
HLA-C*14:02	Phenytoin	DRESS	5.74	Thai	Manuyakorn et al. (2020)
HLA-DRB1*12:02	Carbamazepine	SJS,TEN	28.51	Han Chinese	Shi et al. (2017)
HLA-DRB1*13:02	Allopurinol	SJS,TEN	2.77	Caucasian	Cristallo et al. (2011)
HLA-DRB1*15:02	Allopurinol	SJS,TEN	26.38	Caucasian	Cristallo et al. (2011)
HLA-DRB1*16:02	Phenytoin	SJS,TEN	0.0	Han Chinese	Hung et al. (2010)

MPE, maculopapular exanthema; SCARs, severe cutaneous adverse reactions; SJS, Stevens-Johnson syndrome; TEN, Toxic epidermal necrolysis; DRESS, drug reaction with eosinophilia and systemic symptoms; AGEP, Acute Generalized ExanthematousPustulosis; CF, carrier frequency; HLA, human leukocyte antigen.

## Conclusion

For the past 10 years, our PPM lab has seen an increase in *HLA-B* genotyping, particularly for medications like carbamazepine, allopurinol, abacavir, and nevirapine. HLA-B*15:02, HLA-B*58:01, HLA-B*57:01, HLA-B*13:01, HLA-B*15:21, HLA-B*35:05 alleles are found to be associated with drug-induced hypersensitivity reactions. This study shows that in the future, Thailand will have even more PGx data available for numerous medications. However, it will be hard to predict and avoid serious ADRs if we do not take full advantage of PGx, pharmacovigilance, as well as other biological, clinical, and non-clinical data. As a result, it is critical to create a single framework that incorporates all data in order to provide tailored treatment. It is crucial in countries like Thailand, where there’s already a lot of PGx data.

## Data Availability

The original contributions presented in the study are included in the article/Supplementary Material, further inquiries can be directed to the corresponding author.
