# Supplementary material for: Evolution of HLA-B Pharmacogenomics and the Importance of PGx Data Integration in Health Care System: A 10 Years Retrospective Study in Thailand
Source: Front Pharmacol. 2022 Apr 5;13:866903. doi: 10.3389/fphar.2022.866903 (PMC9016335; doi:10.3389/fphar.2022.866903)
Supplement: Supplementary file 1 [file Table1.docx]

**Supplement table S1** **The allele frequencies and carrier frequencies of *HLA-B* alleles in Thai population (N=12,425)**

| **No.** | **HLA-B alleles** | **Allele frequencies** | **Carrier Frequencies (%)** |
| --- | --- | --- | --- |
| 1 | *HLA-B*07:02* | 0.97 | 1.93 |
| 2 | *HLA-B*07:03* | 0.01 | 0.01 |
| 3 | *HLA-B*07:05* | 2.17 | 4.34 |
| 4 | *HLA-B*07:09* | 0.01 | 0.01 |
| 5 | *HLA-B*07:13* | 0.02 | 0.03 |
| 6 | *HLA-B*07:14* | 0.01 | 0.01 |
| 7 | *HLA-B*07:18* | 0.01 | 0.02 |
| 8 | *HLA-B*07:20* | 0.01 | 0.01 |
| 9 | *HLA-B*08:01* | 0.69 | 1.37 |
| 10 | *HLA-B*08:02* | 0.01 | 0.02 |
| 11 | *HLA-B*08:03* | 0.01 | 0.02 |
| 12 | *HLA-B*08:23* | 0.01 | 0.01 |
| 13 | *HLA-B*08:27* | 0.01 | 0.01 |
| 14 | *HLA-B*08:33* | 0.01 | 0.02 |
| 15 | *HLA-B*08:35* | 0.01 | 0.01 |
| 16 | *HLA-B*13:01* | 6.45 | 12.91 |
| 17 | *HLA-B*13:02* | 1.43 | 2.86 |
| 18 | *HLA-B*13:13* | 0.01 | 0.01 |
| 19 | *HLA-B*13:23* | 0.01 | 0.01 |
| 20 | *HLA-B*13:28* | 0.01 | 0.01 |
| 21 | *HLA-B*13:39* | 0.01 | 0.01 |
| 22 | *HLA-B*14:01* | 0.02 | 0.04 |
| 23 | *HLA-B*14:02* | 0.11 | 0.22 |
| 24 | *HLA-B*14:13* | 0.01 | 0.01 |
| 25 | *HLA-B*15:01* | 1.73 | 3.45 |
| 26 | *HLA-B*15:02* | 7.48 | 14.96 |
| 27 | *HLA-B*15:03* | 0.05 | 0.11 |
| 28 | *HLA-B*15:04* | 0.09 | 0.18 |
| 29 | *HLA-B*15:05* | 0.04 | 0.08 |
| 30 | *HLA-B*15:06* | 0.01 | 0.01 |
| 31 | *HLA-B*15:07* | 0.15 | 0.3 |
| 32 | *HLA-B*15:08* | 0.07 | 0.13 |
| 33 | *HLA-B*15:10* | 0.04 | 0.09 |
| 35 | *HLA-B*15:11* | 0.3 | 0.6 |
| 36 | *HLA-B*15:12* | 0.35 | 0.69 |
| 37 | *HLA-B*15:13* | 0.83 | 1.66 |
| 38 | *HLA-B*15:15* | 0.01 | 0.01 |
| 39 | *HLA-B*15:16* | 0.01 | 0.01 |
| 40 | *HLA-B*15:17* | 0.33 | 0.66 |
| 41 | *HLA-B*15:18* | 0.38 | 0.76 |
| 42 | *HLA-B*15:20* | 0.06 | 0.12 |
| 43 | *HLA-B*15:21* | 0.54 | 1.09 |
| 44 | *HLA-B*15:22* | 0.01 | 0.01 |
| 45 | *HLA-B*15:25* | 1.58 | 3.17 |
| 46 | *HLA-B*15:27* | 0.22 | 0.44 |
| 47 | *HLA-B*15:29* | 0.01 | 0.02 |
| 48 | *HLA-B*15:30* | 0.01 | 0.01 |
| 49 | *HLA-B*15:32* | 0.5 | 1.01 |
| 50 | *HLA-B*15:35* | 0.42 | 0.84 |
| 51 | *HLA-B*15:64* | 0.05 | 0.1 |
| 52 | *HLA-B*15:73* | 0.01 | 0.01 |
| 53 | *HLA-B*15:88* | 0.01 | 0.01 |
| 54 | *HLA-B*18:01* | 3.54 | 7.08 |
| 55 | *HLA-B*18:02* | 0.95 | 1.91 |
| 56 | *HLA-B*18:18* | 0.01 | 0.01 |
| 57 | *HLA-B*27:01* | 0.01 | 0.02 |
| 58 | *HLA-B*27:03* | 0.35 | 0.69 |
| 59 | *HLA-B*27:04* | 1.64 | 3.29 |
| 60 | *HLA-B*27:06* | 1.32 | 2.63 |
| 61 | *HLA-B*27:07* | 0.09 | 0.19 |
| 62 | *HLA-B*27:15* | 0.02 | 0.04 |
| 63 | *HLA-B*27:61* | 0.01 | 0.01 |
| 64 | *HLA-B*27:86* | 0.01 | 0.01 |
| 66 | *HLA-B*35:01* | 1.61 | 3.22 |
| 67 | *HLA-B*35:02* | 0.12 | 0.23 |
| 68 | *HLA-B*35:03* | 1.06 | 2.13 |
| 69 | *HLA-B*35:05* | 1.86 | 3.72 |
| 70 | *HLA-B*35:08* | 0.05 | 0.1 |
| 71 | *HLA-B*35:13* | 0.01 | 0.02 |
| 72 | *HLA-B*35:20* | 0.01 | 0.01 |
| 73 | *HLA-B*35:37* | 0.01 | 0.01 |
| 74 | *HLA-B*35:46* | 0.01 | 0.01 |
| 75 | *HLA-B*35:68* | 0.01 | 0.02 |
| 76 | *HLA-B*37:01* | 0.7 | 1.4 |
| 77 | *HLA-B*37:19* | 0.01 | 0.01 |
| 78 | *HLA-B*38:01* | 0.11 | 0.22 |
| 79 | *HLA-B*38:02* | 3.47 | 6.94 |
| 80 | *HLA-B*38:04* | 0.02 | 0.03 |
| 81 | *HLA-B*38:13* | 0.01 | 0.01 |
| 82 | *HLA-B*38:20* | 0.01 | 0.01 |
| 83 | *HLA-B*38:22* | 0.01 | 0.01 |
| 84 | *HLA-B*38:23* | 0.03 | 0.05 |
| 85 | *HLA-B*38:29* | 0.01 | 0.01 |
| 87 | *HLA-B*39:01* | 0.91 | 1.82 |
| 88 | *HLA-B*39:02* | 0.02 | 0.03 |
| 89 | *HLA-B*39:03* | 0.03 | 0.05 |
| 90 | *HLA-B*39:05* | 0.04 | 0.09 |
| 91 | *HLA-B*39:06* | 0.02 | 0.03 |
| 92 | *HLA-B*39:09* | 1.17 | 2.34 |
| 93 | *HLA-B*39:15* | 0.24 | 0.48 |
| 94 | *HLA-B*39:24* | 0.04 | 0.08 |
| 95 | *HLA-B*39:49* | 0.01 | 0.01 |
| 97 | *HLA-B*40:01* | 8.94 | 17.87 |
| 98 | *HLA-B*40:02* | 1.08 | 2.16 |
| 99 | *HLA-B*40:03* | 0.03 | 0.05 |
| 100 | *HLA-B*40:04* | 0.35 | 0.69 |
| 101 | *HLA-B*40:05* | 0.01 | 0.01 |
| 102 | *HLA-B*40:06* | 1.1 | 2.2 |
| 103 | *HLA-B*40:09* | 0.01 | 0.01 |
| 104 | *HLA-B*40:10* | 0.02 | 0.04 |
| 105 | *HLA-B*40:21* | 0.01 | 0.01 |
| 106 | *HLA-B*40:23* | 0.01 | 0.01 |
| 107 | *HLA-B*40:33* | 0.01 | 0.01 |
| 108 | *HLA-B*40:59* | 0.01 | 0.02 |
| 109 | *HLA-B*41:01* | 0.1 | 0.2 |
| 110 | *HLA-B*41:10* | 0.01 | 0.01 |
| 111 | *HLA-B*41:16* | 0.01 | 0.01 |
| 112 | *HLA-B*42:01* | 0.01 | 0.02 |
| 113 | *HLA-B*44:01* | 0.01 | 0.01 |
| 114 | *HLA-B*44:02* | 0.36 | 0.71 |
| 115 | *HLA-B*44:03* | 4.71 | 9.42 |
| 116 | *HLA-B*44:06* | 0.01 | 0.01 |
| 117 | *HLA-B*44:18* | 0.01 | 0.01 |
| 118 | *HLA-B*44:43* | 0.01 | 0.01 |
| 119 | *HLA-B*44:54* | 0.01 | 0.01 |
| 120 | *HLA-B*45:01* | 0.04 | 0.09 |
| 121 | *HLA-B*45:02* | 0.01 | 0.01 |
| 122 | *HLA-B*46:01* | 12.28 | 24.56 |
| 123 | *HLA-B*46:02* | 0.02 | 0.03 |
| 124 | *HLA-B*46:12* | 0.24 | 0.48 |
| 125 | *HLA-B*46:16* | 0.01 | 0.01 |
| 126 | *HLA-B*47:01* | 0.01 | 0.02 |
| 127 | *HLA-B*47:03* | 0.01 | 0.01 |
| 128 | *HLA-B*48:01* | 0.56 | 1.13 |
| 129 | *HLA-B*48:03* | 0.24 | 0.47 |
| 130 | *HLA-B*48:04* | 0.03 | 0.05 |
| 131 | *HLA-B*48:21* | 0.02 | 0.03 |
| 132 | *HLA-B*49:01* | 0.05 | 0.1 |
| 133 | *HLA-B*49:05* | 0.01 | 0.01 |
| 134 | *HLA-B*50:01* | 0.21 | 0.43 |
| 135 | *HLA-B*50:12* | 0.01 | 0.01 |
| 136 | *HLA-B*51:01* | 3.59 | 7.18 |
| 137 | *HLA-B*51:02* | 1.1 | 2.2 |
| 138 | *HLA-B*51:03* | 0.6 | 1.21 |
| 139 | *HLA-B*51:04* | 0.07 | 0.14 |
| 140 | *HLA-B*51:06* | 0.08 | 0.16 |
| 141 | *HLA-B*51:07* | 0.06 | 0.12 |
| 142 | *HLA-B*51:08* | 0.01 | 0.01 |
| 143 | *HLA-B*51:09* | 0.01 | 0.01 |
| 144 | *HLA-B*51:45* | 0.01 | 0.01 |
| 145 | *HLA-B*52:01* | 2.42 | 4.84 |
| 146 | *HLA-B*52:07* | 0.01 | 0.01 |
| 147 | *HLA-B*52:11* | 0.01 | 0.01 |
| 148 | *HLA-B*52:25* | 0.01 | 0.02 |
| 149 | *HLA-B*53:01* | 0.07 | 0.14 |
| 150 | *HLA-B*53:08* | 0.01 | 0.01 |
| 151 | *HLA-B*53:14* | 0.01 | 0.02 |
| 152 | *HLA-B*53:17* | 0.02 | 0.03 |
| 153 | *HLA-B*54:01* | 1.26 | 2.51 |
| 154 | *HLA-B*54:04* | 0.01 | 0.02 |
| 155 | *HLA-B*54:14* | 0.01 | 0.01 |
| 156 | *HLA-B*54:16* | 0.01 | 0.01 |
| 157 | *HLA-B*55:01* | 0.31 | 0.61 |
| 158 | *HLA-B*55:02* | 1.61 | 3.22 |
| 159 | *HLA-B*55:04* | 0.03 | 0.07 |
| 160 | *HLA-B*55:10* | 0.01 | 0.01 |
| 161 | *HLA-B*55:12* | 0.01 | 0.02 |
| 162 | *HLA-B*55:13* | 0.01 | 0.01 |
| 163 | *HLA-B*55:23* | 0.02 | 0.04 |
| 164 | *HLA-B*55:32* | 0.01 | 0.01 |
| 165 | *HLA-B*55:34* | 0.01 | 0.01 |
| 166 | *HLA-B*55:44* | 0.01 | 0.01 |
| 167 | *HLA-B*56:01* | 0.78 | 1.57 |
| 168 | *HLA-B*56:02* | 0.5 | 1.01 |
| 169 | *HLA-B*56:03* | 0.1 | 0.21 |
| 170 | *HLA-B*56:04* | 0.01 | 0.02 |
| 171 | *HLA-B*56:10* | 0.03 | 0.05 |
| 172 | *HLA-B*56:12* | 0.01 | 0.01 |
| 173 | *HLA-B*56:16* | 0.01 | 0.02 |
| 174 | *HLA-B*57:01* | 1.67 | 3.33 |
| 175 | *HLA-B*57:03* | 0.02 | 0.04 |
| 176 | *HLA-B*57:21* | 0.03 | 0.05 |
| 177 | *HLA-B*58:01* | 8.02 | 16.03 |
| 178 | *HLA-B*58:02* | 0.03 | 0.05 |
| 179 | *HLA-B*58:04* | 0.01 | 0.01 |
| 180 | *HLA-B*58:16* | 0.01 | 0.01 |
| 181 | *HLA-B*67:01* | 0.1 | 0.21 |
| 182 | *HLA-B*73:01* | 0.04 | 0.09 |
| 183 | *HLA-B*78:04* | 0.01 | 0.01 |
| 184 | *HLA-B*78:05* | 0.01 | 0.02 |
| 185 | *HLA-B*81:01* | 0.03 | 0.05 |
| 186 | *HLA-B*82:02* | 0.01 | 0.01 |

CF= carrier frequency, *HLA=Human leukocyte antigen*
